# Unpacking the Mental Health of Nurses during COVID-19: Evidence from Pakistan

**DOI:** 10.3390/jcm10163546

**Published:** 2021-08-12

**Authors:** Xinxing Xu, Faiza Manzoor, Shaoping Jiang, Ayesha Mumtaz

**Affiliations:** 1 Legislation Research Institution of Zhejiang University, Legislation Research Institution of Zhejiang, Zhejiang University Guanghua Law School, Hangzhou 310008, China; xuxinxing@zju.edu.cn; 2 Department of Agricultural Economics and Management, School of Public Affairs, Zhejiang University, Hangzhou 310058, China; faizamanzoor@zju.edu.cn or; 3 Guanghua Law School, Zhejiang University, Zhijiang Road, Hangzhou 310008, China; 4 College of Public Administration, Zhejiang University, Hangzhou 310058, China

**Keywords:** COVID-19 fear, exposure to COVID-19, psychological well-being, social support, nurses, Pakistan

## Abstract

The prime objective of the present study is to test the effect of COVID-19 fear on the mental condition of nurses in the public health sector of Pakistan. This study seeks to measure the psychological distress, anxiety, and psychological well-being of nurses due to fear of COVID-19 and exposure to COVID-19. This research further reveals the moderating role of “social support” in the link between COVID-19 fear, exposure and mental health. Through a convenient sampling technique, 250 sample contributors were chosen from 12 public hospitals. The results were obtained by applying multiple regression and moderation analysis by SPSS and the Hayes process. The outcomes indicated that fear of exposure to COVID-19 affects the mental health of nurses. The findings also discovered that social support is not very constructive in the pandemic. However, we suggested that social support is the best weapon to encourage nurses to relieve their fear and minimize negative emotions.

## 1. Introduction

The coronavirus disease (COVID-19) has affected more than 213 countries and territories around the world with over 28 million cases and almost one million deaths as of mid-September 2020 [1]. The COVID-19 pandemic has disrupted the lives of people all over the world [2,3,4]. The virus is a coronavirus type; there are many different types, and some of them cause disease. A recently detected coronavirus, Severe Acute Respiratory Syndrome Coronavirus (SARS-CoV-2), has triggered a global pandemic of respiratory disease, named COVID-19 [5]. COVID-19 symptoms include cough, chills or fever, breathing trouble or shortness of breath, body or muscle aches, sore throat, loss of scent or taste, diarrhea, headache, tiredness, vomiting or nausea, runny nose or congestion [6]. In most cases, COVID-19 can lead to serious respiratory problems, kidney failure, or death [7].

The world is suffering from COVID-19, but especially low and middle-income countries are facing a lot of uncertainty because COVID-19 is challenging and comes with a lot of uncertainty [8,9]. These countries, such as Indonesia, South Africa, and China, are also forecasted to have more than one million people pushed into extreme poverty as a consequence of COVID-19. When looking at the impact of the pandemic on higher poverty lines, for example, the number of people living on less than USD 3.20 or USD 5.50 per day, more than 100 million people will be pushed into poverty. In developing countries, low-income workers are more likely to lose their jobs as a result of COVID-19 [10]. Pakistan is one of the developing countries that also continues to be affected by the pandemic. The first case of COVID-19 in Pakistan was reported on 26 February 2020 [11]. From 15 to 25 March 2020, the number of cases rose from 53 to 1078. Since then, the number of cases has increased exponentially day by day in different parts of the country. As of 20 July 2020, there were 265,083 confirmed cases in Pakistan, of which 5599 had died [11].

The pandemic affected the economy badly as well. Different firms had to close, and the state hit an upward unemployment rate [11]. Persistent on the rise of cases of COVID-19, limitation of movement, and social contacts, it has been proposed that the negative effects on the economy and everyday life and the volatility of the political climate have an impact on the mental well-being of the population [12]. Besides, the working circumstances for healthcare staff are greatly impaired and the provision of healthcare services has become emotionally challenging due to uncertainty, anxiety, and stigmatization [13]. The medical workforce provides their services in serious circumstances due to the tentative knock of COVID-19, lack of drugs, cases of death, high level of infection, no standardized operating procedures, deficiency of personal protective apparatus, and lockdown situations [14]. Medical staff are typically attributed to traumatic dealings and often experience the death of patients. However, in the recent COVID-19 epidemic, healthcare employees experienced severe and persistent exposure to these causes, contributing to the development of secondary trauma or post-traumatic stress disorder [15]. Healthcare staff employed in emergency rooms are at greater risk of experiencing one or more signs of depression, stress, and anxiety relative to people working in other departments [16,17,18]. People may suffer great empathetic anxiety and distress by watching or frequently hearing about other people’s painful struggles with coronavirus. Therefore, more distal exposure to the pandemic through the media may cause mental health problems [19]. To date, many reports have been published on the primary and clinical treatment of COVID-19 cases [20,21]. Research shows that at the beginning of the outbreak in Wuhan, China, medical personnel faced more serious psychological problems [22].

Anxiety is only an indicator of an underlying illness, when emotions become exaggerated, overwhelming, and disrupt everyday life [23]. Nurses are most likely to become infected with COVID-19 due to a variety of factors, such as poor working conditions, insufficient personal protective equipment (PPE), insufficient staffing, and inadequate safety procedures. The latest studies published about Coronavirus-19, especially in China, have said that the mental condition of nurses and physicians is intensely affected by such a widespread epidemic. Many researchers have examined the trauma of medical staff working in COVID-19 and reported that such personnel face depression, extreme anxiety, insomnia, and suffering due to traumatic situations [24,25,26,27]. According to Labrague et al. [28], psychological distress, professional and organizational turnover would be increased by a rise in fear of COVID-19, and work satisfaction would be decreased. Prior studies suggested that people are facing psychological distress due to the COVID-19 pandemic [29,30,31]. Furthermore, Baldassarre et al. [32] have evaluated and promoted the significance of psychological aspects of stigma and social discrimination (SAD) in pandemic realities and, particularly in the current context of COVID-19/ SARS-CoV-2. The aim of their study was to suggest a coping strategy to face SAD linked to the COVID-19 pandemic outbreak, using coping strategy tackles and solutions from other common contagious diseases.

Literature found that psychological distress was related to self-reported mood conditions and lifestyle variations [33].

For nurses to play valuable roles in this pandemic, preserving their psychological and mental well-being is important. Recent literature, however, found that COVID-19 had a substantial effect on the emotional and psychological well-being of nurses [34,35]. Moreover, many studies have shown a significant and positive relationship concerning COVID-19 fear, exposure to COVID-19 and mental health problems, such as depression, trauma, burnout, and anxiety [36,37,38,39,40]. Besides, the mental condition of frontline nurses working directly in the coronavirus section is deeply affected as they are eyewitnesses to COVID-19 patients suffering and dying [27,41] leading to extreme stress and anxiety.

Psychological stress is a state of emotional distress that is associated with stressors and demands that are difficult to deal with in everyday life. The lack of effective care and the difficulty of identifying the psychological disorder are frustrating for both patients and health professionals. Psychological well-being is a combination of feeling good and effective functioning [42]. The researchers also found that the absence of stress does not necessarily mean that a person has good psychological well-being. High psychological well-being is a feeling of happiness and doing well. People with high psychological well-being report that they are capable, happy, well-supported, and satisfied with life [43]. Research has shown that people with higher psychological well-being are more likely to live healthier and longer, and more likely to have a better quality of life [44]. People are also more likely to enjoy positive psychological well-being if their basic needs are met. Living in a safe area, having enough food, and enough housing are important factors for emotional health.

The conservation of resources (COR) theory is a stress theory [45]. This theory defines the nature of psychological stress and its possible consequences. Traditionally, stress theories have focused on how people view stressful situations as a determining factor in how much stress they will experience [46]. The COR theory claims that stress is not primary or primarily a product of human event assessment, but that it has a central ecological, social, and cultural basis in terms of the requirements for people to acquire and protect the circumstances that ensure their well-being and distance themselves from threats to their well-being [47]. The COR theory claims that stress arises from difficulties in achieving common goals pursued by cultural representatives [48].

Sustainable well-being does not require people to feel upright all the time; experiencing unpleasant feelings is a natural part of life, and the ability to deal with these painful or negative emotions is crucial to long-term well-being. But psychological well-being deteriorates when negative and painful feelings are difficult or long-lasting and disrupt a person’s work in their daily lives [43]. Previous studies have shown positive psychological well-being to ease stress and anxiety and bring happiness and satisfaction to life [49,50,51]. Thus, nursing psychological well-being is a fantastic strategy that could minimize their anxiety level during this pandemic period, thus, for a vigorous work environment, it is necessary to encourage the nursing staff in this pandemic.

**Figure 1:**
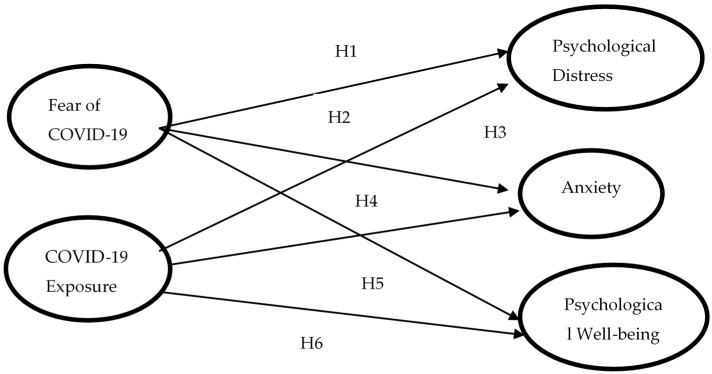
Conceptual Model of the study.

**Figure 2:**
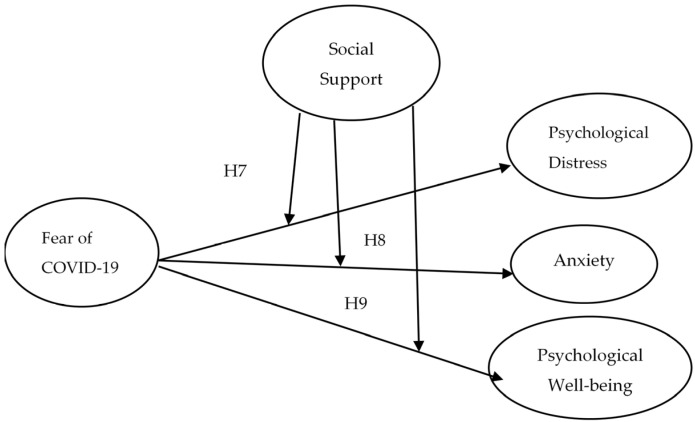
Moderation Model of the study (Fear of COVID-19 and mental health).

**Figure 3:**
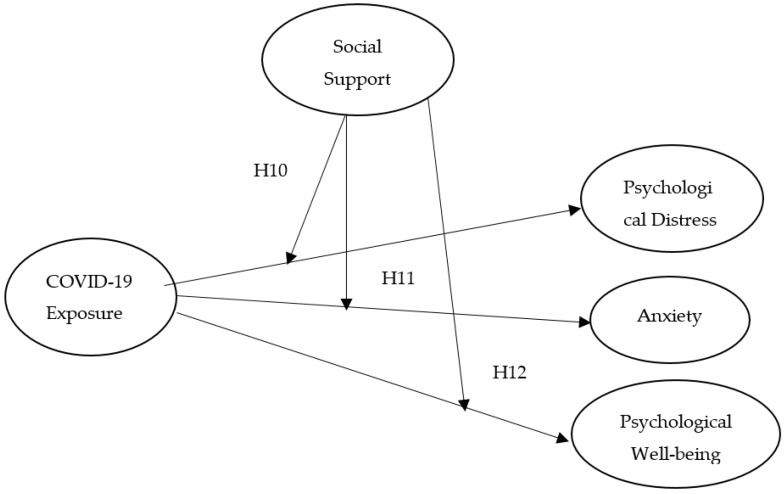
Moderation Model of the study (COVID-19 Exposure and mental health).

The evidence-based assessments on mental health, fear, and exposure to COVID-19 have been quite limited. However, in Pakistan, it is not clear whether the psychological problems of nurses are serious. Therefore, it is extremely important to understand the mental health of nurses, especially in the face of public health emergencies, such as COVID-19. Based on the above-mentioned loopholes in the existing research, the objectives of this study are:

1. Assess the impact of COVID-19 fear and exposure on mental health, such as the level of anxiety, psychological distress, as well as psychological well-being among nurses in Pakistan (see Figure 1);
2. Identify the moderating effect of social support on the association of COVID-19 fear and the nurse’s mental condition (see Figure 2);
3. Examine the moderating influence of social support on exposure to COVID-19 and nurses’ mental health (see Figure 3).

Besides, based on the above arguments we posit hypotheses that:

**Hypothesis** **1** **(H1).** *Fear of COVID-19 has a positive relationship with the psychological distress of nurse’s staff.*

**Hypothesis** **2** **(H2).** *COVID-19 exposure has a positive correlation with the psychological distress.*

**Hypothesis** **3** **(H3).** *Fear of COVID-19 is positively related with the nurses’ anxiety level.*

**Hypothesis** **4** **(H4).** *COVID-19 exposure has a positive relationship with the anxiety level of nurses.*

**Hypothesis** **5** **(H5).** *Fear of COVID-19 has a negative relationship with psychological well-being (positive attitude of nurses can minimize fear of COVID-19).*

**Hypothesis** **6** **(H6).** *COVID-19 exposure has a negative association with the psychological well-being of nurses.*

### Role of Social Support

Social support means having friends and others, such as supervisors, leaders, colleagues, and family, to turn to in times of crisis or need to provide a wider perspective and an optimistic self-image [52].

Social help increases the quality of life and protects against traumatic events life [53]. Potential mechanisms are required to minimize the negative effects of COVID-19 on frontline healthcare personnel. One probable technique to decrease the related risk of mental health issues is to appropriately prepare the staff for work and its associated difficulties [54]. The family, friends, and supervisors must fully assess what nurses are facing during this pandemic. Supervisors or leaders must provide support without false reassurance and euphemisms. Likewise, top management should encourage and support them to make ethically stimulating decisions in the current pandemic, and allow them to openly discuss the emotional and social challenges they are facing during patient care. The leader’s support makes it possible to reduce their fear of COVID-19 and to protect healthcare workers’ or nurses’ mental health [55,56]. Moreover, family and friends’ encouragement and support also make nurses enthusiastic about their duties and responsibilities [57,58]. Past studies have shown that positive social support and job satisfaction have a significant association [59,60]; they also reduce anxiety, burnout, and fear of COVID-19 [61,62], and the moderating effect of social support on the stress–burnout relationship [63]. Therefore, in this study, we assessed the moderating influence of social support on the association of fear of COVID-19, exposure to COVID-19, and mental health (Figure 2 and Figure 3). Thus, we assume that:

**Hypothesis** **7** **(H7).** *Social support may moderate the association among fear of COVID-19 and psychological distress of nurses (i.e., when social support is strong, fear of COVID is low).*

**Hypothesis** **8** **(H8).** *Social support may moderate the correlation between fear of COVID-19 and the anxiety level among nurses (i.e., when social support is high, the effect of fear of COVID-19 is very low).*

**Hypothesis** **9** **(H9).** *Social support may moderate the connection between fear of COVID-19 and psychological well-being of nurses (i.e., COVID-19 fear is very low with high social support).*

**Hypothesis** **10** **(H10).** *Social support may moderate the association among COVID-19 exposure and psychological distress of nurses.*

**Hypothesis** **11** **(H11).** *Social support may moderate the correlation between COVID-19 exposure and the Anxiety level among nurses.*

**Hypothesis** **12** **(H12).** *Social support may moderate the connection between COVID-19 exposure and psychological well-being of nurses.*

## 2. Material and Methods

### 2.1. Research Sample and Data Collection

The key purpose of this study is to examine the nurse’s fear of COVID-19, exposure to COVID-19, and their mental condition with the inclusion of social support as a moderator. This study was conducted in the twelve public health sectors of Khyber Pakhtunkhwa (KPK) and Punjab Provinces of Pakistan. The participants of this study were nurses who are currently working in public hospitals in KPK and Punjab provinces. The time slot of data collection for this study was May 2020–June 2020. Most of the women nurses were participants in the study. In the country, female nurses are higher than males and the concept of care is associated with women in our society. Therefore, patients prefer female nurses over male nurses. The number of men entering the nursing profession remains very low. Nurses are primary care providers and are responsible for patients’ care. For centuries, the nursing profession has been recognized as a profession only for women [64]. Questionnaires were circulated among the participants and consisted of all study variables and socio-demographic information. Questions of the study variables are previously validated questionnaires that exist in the literature, which were already used in studies of various cultures and countries. These are all validated instruments. A validated questionnaire refers to a questionnaire/scale that has been designed for use among prospective respondents [65]. Originally, 300 above questionnaires were distributed, and only 250 were returned.

### 2.2. Instrumentation

As shown in the Table 1, the main instruments of the study were fear of COVID-19 that was measured by the seven-item scale; this scale was adopted by the study of Ahorsu et al. [66]. An example item is “How emotionally does your disease affect you?” COVID-19 exposure is measured by two items, which were adopted by the study of Guo et al. [67]. Example items are ”Exposure to the COVID-19 pandemic through watching or using the media” was answered on a four-point rating scale for frequency: (very frequent, often, some, no exposure) and possibly having suffered or suffering from COVID-19, or someone in the family, or neighbourhood, or among friends”, with “1” for COVID-19 of self, a member of the family, a friend, or someone in the neighbourhood, and “0” referring to no exposure). To measure the nurse’s anxiety with the five-item scale, settled by Bostan et al. [68]. An example item is “I’m concerned about getting sick with COVID-19”. Psychological distress is quantified by the five-item scale which was developed by Cavanagh et al. [69], also used in previous literature [14]. Psychological well-being is measured by the five-item scale of the World Health Organization. This scale was adopted by the study by De Wit et al. [70]. Social support is measured by a 13-item scale by Andrews, et al. [71] and Kaniasty, et al. [72]. An example item is “My family is very supportive of me”. All items are rated on a five-point Likert scale, i.e., 5 strongly agree to 1 strongly disagree. The Cronbach alpha reliability value of fear of COVID-19 and psychological distress is 0.854 and 0.795, respectively. Likewise, Cronbach α for COVID-19 exposure and anxiety conditions was 0.744 and 0.913 and for psychological well-being was 0.944. For the scale of social support, the alpha coefficient was 0.983. The values of alpha reliability met the cutoff criteria [73,74,75].

### 2.3. Data Analysis

Firstly, to assess the hypotheses of the study, a multiple regression model was employed through IBM-SPSS 25 version [76]. Our study model (see Figure 1) has two predictor variables (fear of COVID-19 and exposure to COVID-19) and three outcome variables (psychological distress, anxiety, and psychological well-being). To estimate the relative influence of the explanatory variables on the particular outcome, multiple linear regression analysis is ideal [77]. As a predictive analysis, multiple linear regression is applied to describe the association between one continuous outcome variable and two or more predictor variables [78]. The purpose of this analysis is to predict the value of a variable based on the value of two or more other variables. 

Furthermore, in the present study, social support (SS) was employed as a moderator variable. A moderation analysis is a form of regression analysis that describes the effect of the explanatory variable on the outcome variable by or under the control of a moderator, which is a third variable [79]. Generally, moderator effects are specified by the interaction of X and M in explaining Y.

For the study, the Hayes process [80] through the software IBM-SPSS version 25 has been used to test the moderation hypotheses. The Hayes process is considered a more powerful and effective process than its alternatives [81], and 5000 bootstrapping-based resamples have been selected. The results of moderation analysis in comparison to multiple regression are a little different.

## 3. Results

### 3.1. Descriptive Statistics

Socio-demographic information, such as gender, age, and education, are measured by categorical variables, such as age (1= “20–25”, 2 = “26–30” years, 3 = “31–35” years, 4 = “36–39” years, and 5 = “≥40 years”), gender (1 = male and 2 = female), and education is measured by (1 = “BS nursing”, 2 = “graduate”, and 3 = “undergraduate”). Most of the participants were female (240) with 96%. Most of them were in the 30–40-year age group. Most respondents had a BS nursing degree (191) with 76.4%. The outcomes of the frequency analysis are stated in Table 2.

Table 3 displays the descriptive statistics, correlations between the study variables, and alpha values of the scales. Fear of COVID-19 has a positive correlation with psychological distress (r = 0.585), and the anxiety condition of nurses (r = 0.224). Furthermore, fear of COVID-19 has a negative correlation with psychological well-being (r = −0.466), social support (r = −0.075), and COVID-19 exposure (r = 0.093). Harman’s single factor has been used to test measurement biases [73,82], which shows that data does not suffer from the common method bias issue as the percentage of variance described by a single factor is 30.05 percent, which is less than 50%. The study data can therefore be accepted as valid [83,84].

### 3.2. Regression Results and Interpretation

Table 4 illustrates the positive association between fear of COVID-19 and psychological distress (estimated coefficient = 0.532, *p* < 0.01) and COVID-19 exposure and psychological distress (coefficient = 0.096, *p* < 0.1). Thus, hypothesis 1 and 2 are accepted. The value of R-squared shows that 34 percent of the variation in psychological distress is due to explanatory variables. The results exposed an intense and wide-range of psychological effects on nurses during the outbreak of the virus.

Furthermore, the results in Table 4 show that fear of COVID-19 exposure and the level of anxiety of nurses have a positive and significant relationship. According to the analysis, the *p*-value is far less than 0.05. Therefore, findings demonstrate that COVID-19 fear and exposure to COVID-19 is a cause of anxiety. Anxiety is only an indicator of an underlying sickness, when feelings become excessive, tiring and interfere with daily life. The R-squared value implies that 0.075 percent variation in anxiety was due to fear of COVID-19 and exposure to COVID-19. The estimated coefficient is positive, which shows that fear of COVID-19 and the anxiety condition of nurses have a significant and positive association. Consequently, we accepted hypothesis 3 and 4, respectively. 

Moreover, this table displays the fear of COVID-19 and psychological well-being results, i.e., (β = −0.551, *p* < 0.01, *t* = −8.24) as well as COVID-19 exposure and psychological well-being results (estimated coefficient = −0.044, *p* > 0.05). The value of R-squared means that 21 percent variation in psychological well-being is occurring due to independent variables (fear of COVID-19 and COVID-19 exposure). Further, the *p*-value of fear of COVID-19 and psychological well-being is 0.000, which is far less than 0.05, and the value of t-statistics is also above the cutoff value of 1.96 [75,85,86]. Further, estimated coefficients indicate COVID-19 exposure and psychological well-being have a negative and insignificant association, hence supporting hypothesis 5 and 6 respectively. Psychological well-being is the ability of humans to cope with human anxiety and depression. The results show that nurses have strong psychological well-being during an outbreak.

### 3.3. Moderation Effect

**Figure 4:**
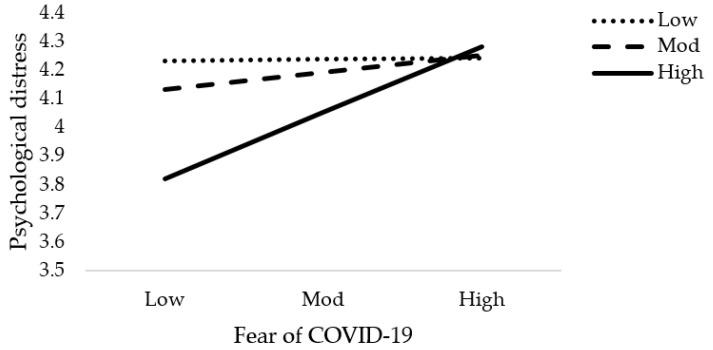
Interactive effect of fear of COVID-19 and social support on psychological distress4. Discussion.

**Figure 5:**
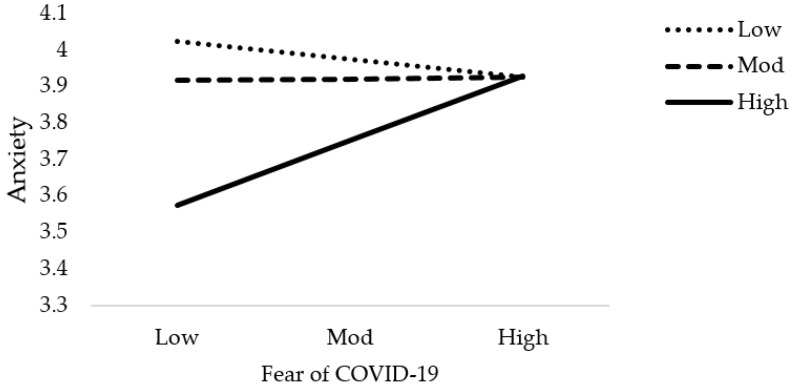
Interactive effect of fear of COVID-19 and social support on Anxiety.

**Figure 6:**
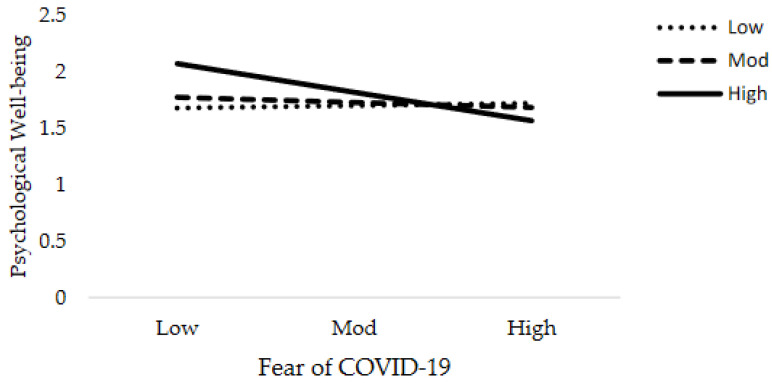
Interactive effect of fear of COVID-19 and social support on Psychological Well-being.

**Figure 7:**
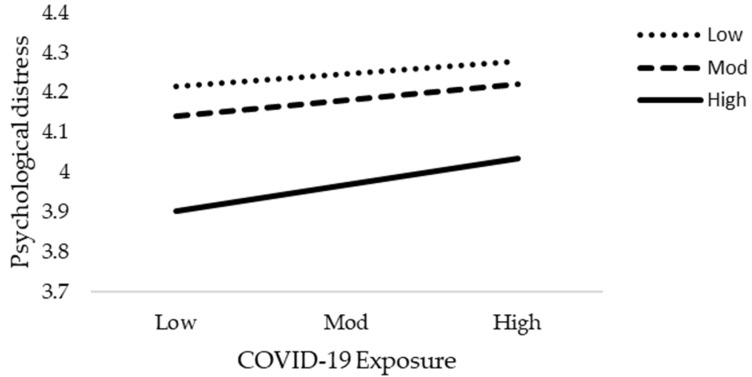
Interactive effect of COVID-19 Exposure and social support on psychological distress.

**Figure 8:**
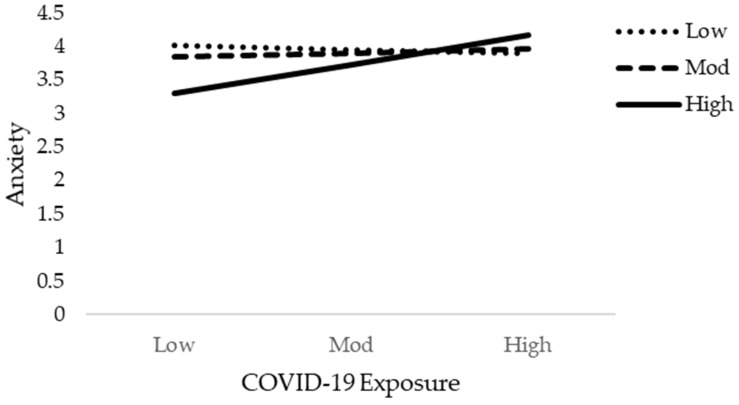
Interactive effect of COVID-19 Exposure and social support on Anxiety.

**Figure 9:**
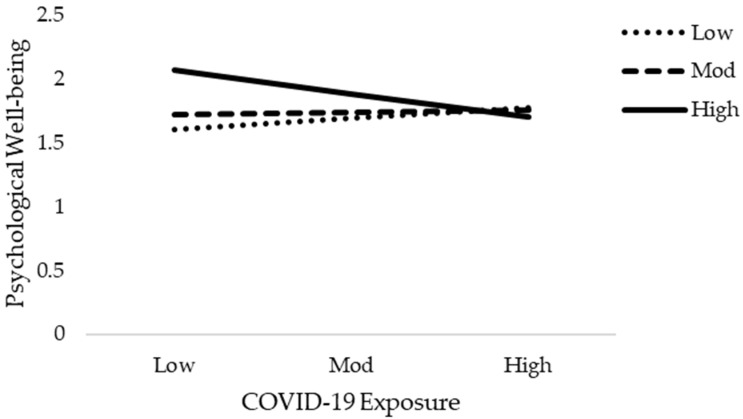
Interactive effect of COVID-19 Exposure and social support on Psychological Well-being.

The results of moderation analysis in comparison to multiple regression are a little different. Table 5 demonstrates the outcome of the moderation effect of social support on fear of COVID-19 as well as COVID-19 exposure and mental health. Table 5 reveals the interaction effects (Int-1) of all six models (2a–2f). This study anticipated that the effect of Int-1 (FOC-19*SS) on psychological distress, anxiety level of nurses, and psychological well-being are significant. The lower limit confidence interval (LLCI) and upper limit confidence interval (ULCI) do not contain zero. It is evident that social support does moderate the association of fear of COVID-19 with psychological distress, level of anxiety, and psychological well-being. Furthermore, Int-1 (CovE*SS) has an insignificant effect on psychological distress, but has a significant effect on anxiety level and psychological well-being. Figure 4, Figure 5, Figure 6, Figure 7, Figure 8 and Figure 9 show the interaction plots for all six relationships. Figure 4, Figure 5 and Figure 6 illustrate the relationship between the interaction between social support and fear of COVID-19 on psychological distress, anxiety level, and psychological well-being. Figure 7, Figure 8 and Figure 9 explain the association between the interaction between social support and COVID-19 exposure on psychological distress, anxiety level, and psychological well-being. Strong social support, low fear of COVID-19 and less exposure to COVID-19, low psychological distress, and anxiety. Under strong social support, there would be no significant correlation between fear of COVID-19, exposure to COVID-19, and psychological distress as well as anxiety. In other words, the individuals’ slops of the two lines suggested that fear of COVID-19 had weak productive power under strong social support, but not quite so under weak social support.

The condition of the nurses’ mental health during COVID-19 was assessed by using fear and exposure to COVID-19 as independent variables and social support as a moderator. We found a positive and significant association between the fear of COVID-19 and the psychological distress of nurses. The results of this study reinforce previous studies underlining New York health workers’ stress due to the outbreak of COVID-19 [87]. These results are also compatible with the previous study findings of Qiu et al., Li et al., and Zhang et al. [88,89,90]. Furthermore, the outcome of the fear of COVID-19 has a significant and positive relationship with the level of anxiety among nurses. These outcomes are consistent with the findings of the previous study [91,92,93,94,95]. Likewise, the results of COVID-19 fear and psychological well-being have a negative and significant association. Psychological well-being states the inter-and intra-individual levels of proper functioning, which can include relationships with others and self-esteem, including a sense of mastery and self-improvement. Subjective well-being signifies aspects of the effect of life satisfaction decisions [96]. The results of the fear and psychological well-being are aligned with the past studies of Asad et al. [97] and Dumalaon-Canaria et al. [98].

Additionally, this study has shown that the findings of the COVID-19 exposure have a significant and positive relationship with the psychological distress and anxiety levels of nurses. These findings are in line with the results of previous research studies [40,67]. Furthermore, the findings of COVID-19 exposure and psychological well-being have a negative and insignificant association. These results are supported by the findings of a previous research study [99]. Moreover, the study explored the moderating role of social support between fear of COVID-19 and mental health (Figure 2), as well as COVID-19 exposure and mental health (Figure 2), which is very limited and nearly non-existent. However, the present study assessed this break and found that social support had a moderated effect on the relationship between COVID-19 fear and mental conditions such as psychological distress and level of anxiety among nurses. As well, social support has no moderate influence on the association between COVID-19 exposure and psychological distress; whereas, social support had a moderated effect on the association between COVID-19 exposure and anxiety level as well as psychological well-being. Theoretically, previous literature has shown that social support and fear of any situation have a negative association [100,101,102]. In contrast, the analysis of this study indicated that social support during this pandemic duration is very low in Pakistan. The study proved that social support for nurses from family, friends, and others is not encouraging. In short, in the pandemic situation, social support was rather poor. Social support can help to overcome setbacks, solve problems, increase self-esteem, and even cope with health problems and stress. People who feel they have the social support they need tend to be less stressed. It is possible that a lack of social support and feelings of loneliness can make mental health or substance use problems more vulnerable, such as depression.

COVID-19 is a world-wide deadly virus that affects human mental health. Pakistan is included in one of the developing countries, facing many challenges in this pandemic era [103]. The number of masks, sanitizers, and virus testing kits is very low and the population of the country is very high. In this scary scenario, nurses, as well as all health workers, should be encouraged by the hospital administration, family, friends, colleagues, supervisors, and the government. This kind of support could help them to reduce their anxiety levels and psychological distress. Furthermore, the provision of basic facilities for nurses plays a key role in boosting their enthusiasm for work, and positive social support and encouragement would help to mitigate their fear. It is hoped that this research will play a significant role in the literature of the healthcare field. Besides, more attention should be paid by the government to the maintenance level of the country’s healthcare sector, and top management should provide training opportunities related to the existing epidemic, provide personal protective apparatus, share correct information, give gratitude, and inspire them with some financial and career development incentives.

Our study also has some limitations, which propose inquiries for future research. Firstly, the primary data has been gathered from the nurses; a future study could use data from all medical personnel, such as physicians/doctors, epidemiologists, psychiatrists, and other medical administration. Secondly, this study is limited to a few hospitals in the context of two provinces of Pakistan. Future studies may be performed in the other medical centers of the country’s remaining provinces. Moreover, the present study is limited to one developing country. Future studies are strongly suggested for other emerging, low-and middle-income nations. A cross-sectional study design is used for data collection. To avoid the vagueness of a causal relationship, a future study may use a longitudinal study method to present the study model.

Though the COVID-19 outbreak appears to have an immediate impact on nurses’ mental health, future research should focus on assessing nurses’ symptoms of depression, stress, and anxiety in the aftermath of the COVID-19 pandemic in order to contrast and compare the results with the results of our research.

In addition, it is well known that the spread of disease is influenced by people’s willingness to adhere to preventive public health practices, which are often associated with public perceptions of risk. As part of a future study, it will be possible to assess the public perception of the risk of COVID-19 around the world by using national data.

## 4. Conclusions

To conclude, our results suggest that during the epidemic, nurses experienced psychological stress and anxiety. Their mental health was cataclysmic. They acquired rare social support from family, friends, colleagues, and top management. Therefore, there is a strong need to overcome their fear with a positive and encouraging attitude in the social circle. It is proposed to create a safe and efficient work environment as this can enhance a personal sense of control and maximize the resilience of the nurse during a pandemic. Hospital management should provide psychological support, financial support, on-the-job training, and supportive supervision to nurses and other emergency personnel who directly deal with COVID-19 patients. To improve the overall mental health of nurses, counselling and psychological support should be actively pursued. In addition, it is also prudent for healthcare management to share accurate and reliable information on managing stress, reducing burnout, and increasing nursing staff’s resilience during a crisis of this magnitude. Providing nurses with up-to-date and accurate information related to COVID-19 can reduce the fear and negative emotions associated with the disease.

Furthermore, health policy makers must develop communication skills and actively manage media relations, and strengthen the capacity of essential public health services to respond to emergencies. Hospitals must be designated to receive COVID-19 patients and prepare to mobilize intensive care and emergency unit (ICU) capacity. Likewise, COVID-19 response services must be organized and expanded close to home and maintain the continuity of essential services by unlocking the COVID-19 response capabilities. The physical health of frontline healthcare workers must be protected. Finally, train, repurpose and mobilize the health workforce according to priority services.

## Figures and Tables

**Table 1:** Sources of Measurement Instruments.

Variable	No of Items	Source
Fear of COVID-19	07	Ahorsu et al. [66]
COVID-19 Exposure	02	Guo et al. [67]
Anxiety	05	Bostan et al. [68]
Psychological distress	05	Cavanagh et al. [69]
Psychological well-being	05	De Wit et al. [70]
Social support	13	Andrews et al. [71] and Kaniasty et al. [72]

**Table 2:** Demographic valuation.

Gender	No.	Percent	Age	No.	Percent	Education	No.	Percent
Male	10	4.0	20–25	6	2.4	BS Nursing	191	76.4
Female	240	96	25–30	40	16	Graduate	59	23.6
Total	250	100	31–35	45	18	Total	250	100
			36–40	111	44.4			
			40 or above	48	19.2			
			Total	250	100			

**Table 3:** Descriptive Statistics, Reliabilities and Correlation Matrix.

Parameters	Mean	St.d	1	2	3	4	5	6
Fear of COVID-19	4.116	0.557	**(0.854)**					
COVID-19 Exposure	1.828	0.547	0.093	**(0.744)**				
Psychological Distress	4.151	0.511	0.585 **	0.107	**(0.795)**			
Anxiety	3.880	0.873	0.224 **	0.184 **	0.404 **	**(0.913)**		
Psychological well_being	1.761	0.658	−0.466 **	−0.038	−0.463 **	−0.234 **	**(0.944)**	
Social support	4.095	0.658	−0.075	−0.038	0.216 **	0.133 *	0.135 *	**(0.983)**

Note: Correlation is significant at the * *p* < 0.05, ** *p* < 0.01. Alpha values are in Parentheses; *n* = (250).

**Table 4:** Regression Analysis of COVID-19 Fear & Exposure and Mental Health.

Variables	Coefficient	Std. Error	*t*-Statistic	*p*-Value	R-Square
DV: Psychological distress					
Constant	1.814	0.232	7.82	0.000	0.344
Fear of COVID-19	0.532	0.047	11.227	0.000	
COVID-19 Exposure	0.096	0.057	1.695	0.091	
DV: Anxiety					
Constant	1.762	0.471	3.744	0.000	0.075
Fear of COVID-19	0.326	0.096	3.392	0.001	
COVID-19 Exposure	0.254	0.094	2.685	0.008	
DV: Psychological Well-being					
Constant	4.009	0.327	12.259	0.000	0.217
Fear of COVID-19	−0.551	0.066	−8.24	0.000	
COVID-19 Exposure	−0.044	0.074	−0.592	0.554	

Note: DV = Dependent variable, *p*-Value = Probability value.

**Table 5:** Moderating effects of Social Support.

	Coeff	SE	T	*p*	LLCI	ULCI
**DV: Psychological distress:**						
Constant	5.478	0.424	12.903	0.000	4.642	6.314
FOC-19	−0.284	0.102	−2.761	0.006	−0.483	−0.081
Social Support	−1.193	0.131	−9.111	0.000	−1.451	−0.935
**Interaction-I**	0.272	0.031	8.609	0.000	0.210	0.334
**DV: Anxiety:**						
Constant	6.027	0.983	6.130	0.000	4.091	7.964
FOC-19	−0.475	0.236	−2.006	0.045	−0.941	−0.0087
Social Support	−1.211	0.303	−3.995	0.000	−1.809	−0.614
**Interaction-I**	0.274	0.073	3.737	0.000	0.129	0.418
**DV: Psychological Well-being:**						
Constant	−0.156	0.632	−0.247	0.804	−1.403	1.089
FOC-19	0.436	0.152	2.864	0.004	0.136	0.736
Social Support	1.408	0.195	7.216	0.000	1.024	1.792
**Interaction-I**	−0.330	0.047	−6.995	0.000	−0.422	−0.237
**DV: Psychological distress:**						
Constant	4.242	0.380	11.138	0.000	3.492	4.992
COVID-19 Exposure	0.036	0.126	0.291	0.771	−0.211	0.285
Social Support	−1.167	0.151	−1.118	0.264	−4.63	0.127
**Interaction-I**	0.024	0.050	0.476	0.634	−0.075	0.123
**DV: Anxiety:**						
Constant	5.523	0.631	8.741	0.000	4.278	6.767
COVID-19 Exposure	−0.495	0.209	−2.368	0.018	−0.907	−0.083
Social Support	−1.107	0.249	−4.448	0.000	−1.598	−0.617
**Interaction-I**	0.343	0.083	4.120	0.000	0.179	0.508
**DV: Psychological Well-being:**						
Constant	0.495	0.492	1.005	0.315	−1.475	1.465
COVID-19 Exposure	0.374	0.163	2.293	0.022	0.052	0.695
Social Support	0.617	0.194	3.180	0.001	0.235	1.000
**Interaction-I**	−0.183	0.065	−2.822	0.005	−0.311	−0.055

Note: FOC-19 = Fear of COVID-19; DV = Dependent variable.

## Data Availability

The data of this study will be available from the corresponding author (A.M.) upon request.
